# PPO-Based Reinforcement Learning Control of a Flapping-Wing Robot with a Bio-Inspired Sensing and Actuation Feather Unit

**DOI:** 10.3390/s26031009

**Published:** 2026-02-04

**Authors:** Saddam Hussain, Mohammed Messaoudi, Muhammad Imran, Diyin Tang

**Affiliations:** 1 School of Automation Science and Electrical Engineering, Beihang University, Beijing 100191, China; 2 Department of Mathematics and Statistics, College of Science, Imam Mohammad Ibn Saud Islamic University (IMSIU), Riyadh 11432, Saudi Arabia; mmessaoudi@imamu.edu.sa; 3 School of Computer and Information Engineering, Henan University, Kaifeng 475000, China; imran@henu.edu.cn

**Keywords:** PPO, reinforcement learning control, bio-inspired, flow sensing, flapping wing, flying robots

## Abstract

Bio-inspired flow-sensing and actuation mechanisms offer a promising path for enhancing the stability of flapping-wing flying robots (FWFRs) operating in dynamic and noisy environments. This study introduces a bio-inspired sensing and actuation feather unit (SAFU) that mimics the covert feathers of falcons and serves simultaneously as a distributed flow sensor and an adaptive actuation element. Each electromechanical feather (EF) passively detects airflow disturbances through deflection and actively modulates its flaps through an embedded actuator, enabling real-time aerodynamic adaptation. A reduced-order bond-graph model capturing the coupled aero-electromechanical dynamics of the FWFR wing and SAFU is developed to provide a physics-based training environment for a proximal policy optimization (PPO) based reinforcement learning controller. Through closed-loop interaction with this environment, the PPO policy autonomously learns control actions that regulate feather displacement, reduce airflow-induced loads, and improve dynamic stability without predefined control laws. Simulation results show that the PPO-driven SAFU achieves fast, well-damped responses with rise times below 0.5 s, settling times under 1.4 s, near-zero steady-state error across varying gust conditions and up to 50% alleviation of airflow-induced disturbance effects. Overall, this work highlights the potential of bio-inspired sensing-actuation architectures, combined with reinforcement learning, to serve as a promising solution for future flapping-wing drone designs, enabling enhanced resilience, autonomous flow adaptation, and intelligent aerodynamic control during operations in gusts.

## 1. Introduction

Flight within the atmospheric boundary layer exposes flapping-wing flying robots (FWFRs) to highly unpredictable and energetic turbulent motions. Sharp gusts occurring in this zone can instantly disrupt attitude and velocity, pushing the aircraft away from its intended trajectory. Such environments require FWFRs to be equipped with resilient gust-alleviation systems supported by sophisticated control algorithms to preserve stability [1,2,3].

Several traditional disturbance control strategies for drones have been extensively investigated till date. Reference [4] presented a gust-alleviation technique that leverages pressure sensors to capture instantaneous flow conditions, providing phase-advance disturbance information unavailable to typical inertial sensing. The outputs from two sensors mounted on the wing were used to drive a PID controller designed to reduce gust-generated rolling moments. Their simulations confirmed better disturbance suppression when flow data were included. However, the method has been validated solely on fixed-wing unmanned aerial vehicles (UAVs), leaving its relevance to flapping-wing systems uncertain.

Synthetic jet actuators have recently been shown to enhance flying robots’ performance by lowering drag and suppressing gust-driven oscillations, resulting in more stable flight behavior [5]. Likewise, vortex generators have demonstrated effectiveness in easing wing loads and postponing wing-tip stall during turbulent or high-speed maneuvers [6]. Yet, despite these promising outcomes, such technologies have been explored almost exclusively on fixed-wing configurations, with little understanding of how they might translate to FWFRs.

Another contribution in [7] proposed a turbulence-mitigation approach aimed at reducing gust-detection latency and improving the reaction speed of a UAV’s onboard control system. By applying advanced signal-processing techniques to micro-electromechanical systems (MEMS) gyroscope measurements, the authors enhanced the vehicle’s stability during small-scale gust disturbances.

Several turbulence-resilient biomimetic UAV architectures have been proposed in the recent literature taking lead from natural flyers [8,9,10,11,12]. A notable example is the “fly-by-feel” method in [13], which equips flapping-wing drones with strain sensors embedded along the wings to mimic mechanosensory feedback in insects. Reinforcement learning is used to decode these strain fields, enabling real-time estimation of attitude and airflow independent of inertial sensing. Validation through simulations and flight tests in 3–7 m/s gusts demonstrated robust, adaptive control. Nonetheless, the technique has not been evaluated under higher turbulence intensities exceeding 7 m/s.

Encouraged by the behavior of avian feathers, the study in [14] proposed a flapping-wing UAV concept equipped with artificial feathers to alleviate gust loads in turbulent flow conditions. Their results showed that feathered wings handled gust disturbances more effectively than conventional rigid-wing configurations. This concept was further developed in [15], where an H-infinity based controller was implemented to stabilize the naturally unstable feathered wing. However, the resulting control input efficiency remained low. Later in [16], a composite controller approach along with observer design was presented, which achieved both robustness and control input efficiency.

Beyond bio-inspired gust-alleviation mechanisms, considerable research has explored gust mitigation in FWFRs through both linear and nonlinear control strategies [17,18,19,20]. One recent investigation examined the problem of post-disturbance recovery in flapping-wing micro aerial vehicles (FWMAVs) experiencing severe attitude deviations caused by aggressive maneuvers and turbulent winds. The authors developed a reinforcement-learning-based controller capable of restoring stable flight while suppressing excessive angular accelerations. To further enhance stability after recovery, they proposed a hybrid RL–PD framework. Simulation outcomes demonstrated that both the standalone RL controller and the hybrid strategy performed robustly under highly demanding flight scenarios, while real-world experimental validation was identified as an important future step [21].

Reference [22] presents a layered model predictive control (MPC) architecture that integrates a quasi-steady aerodynamic estimator with dual control layers to enhance FWMAV performance. Simulation studies demonstrate improved speed and accuracy over PID and traditional MPC when subjected to gusts. Yet, the controller degrades under higher gust intensities, underscoring its limited robustness in severe disturbance environments.

In [23], the authors employed an LQR controller for trajectory tracking in an insect-scale flapping-wing robot flying at speeds up to 25 cm/s. However, the study considered only mild disturbances and did not investigate gust-rejection capability. Subsequent work by Bhatia et al. [24] applied LQR to regulate the attitude of a FWFR in turbulent airflow. Although the platform exhibited open-loop instability, their results demonstrated that closed-loop LQR control was crucial for maintaining attitude stability and preserving the flight envelope under gust excitation, with effective disturbance rejection reported up to 3 m/s.

Zheng et al. [25] developed a tailless flapping-wing robot incorporating three independently actuated wing pairs and bio-inspired passive elastic legs, allowing it to switch seamlessly between ground locomotion and flight. Control was achieved through a cascaded PID framework integrated with an SE (3) geometric tracking scheme, enabling stable hovering and precise trajectory tracking. Although the yaw degree of freedom remained underactuated, the robot exhibited dependable stability and maneuverability. While the experiments did not include high-intensity gusts, the system showed adequate resilience to small external disturbances.

**Figure 1:**
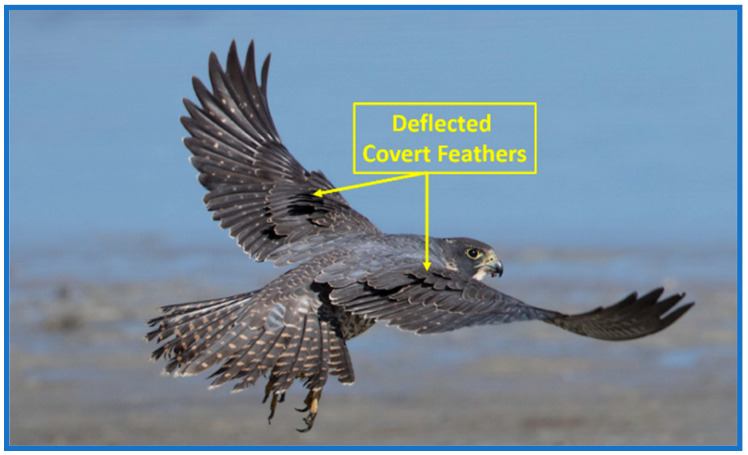
Falcon’s covert feathers deflect in response to gust [28].

The surveyed conventional and bio-inspired gust alleviation designs along with reviewed controllers, work reasonably well for minor disturbances and low wind speeds; however, they tend to lose performance under stronger winds. The main reason is that the gust rejection in flapping-wing systems is often treated as an isolated control challenge, with limited reliance on adaptive aerodynamic features. Survey findings in [26,27] similarly conclude that single-layer controllers struggle to maintain stability in the highly nonlinear and rapidly varying flow environments encountered by FWFRs. These studies emphasize the need for active aerodynamic components; such as feather-inspired mechanisms or multi-tiered control frameworks to achieve dependable gust-mitigation performance. This gap has motivated researchers to examine natural flyers, whose evolutionary traits offer valuable insights into gust handling. Observations show that birds frequently transition into intermittent flight modes under turbulent conditions, briefly gliding or hovering while their covert feathers deploy passively to counter turbulence and buffer sudden gust loads [27,28]. One of the falcon’s flights during gusts shows this behavior, as illustrated in Figure 1.

Authors in [29,30] demonstrate that flapping-wing drones and ornithopters equipped with biomimetic feather structures exhibit inherent dynamic instability when exposed to gusts, making active control essential for maintaining stable and robust flight in turbulent environments. Building upon these findings, the present study introduces a bio-inspired sensing and actuation feather unit (SAFU) comprising electromechanical feathers (EFs) and augmented with a Proximal Policy Optimization (PPO) based reinforcement learning (RL) control framework for a flapping-wing flying robot (FWFR). Unlike conventional gust mitigation strategies that are limited to small-scale disturbances and rely on traditional optimal or robust control laws, the proposed biomimetic SAFU design combined with a learning-based framework does not depend on predefined control rules. Instead, it learns optimal actuator actions directly through interaction with the gust-disturbed environment. The main contributions of this research are appended below:

- A falcon-inspired flow-control design, consisting of EFs integrated into a SAFU and modeled through a reduced-order bond-graph formulation.
- A PPO-based RL controller that learns gust-mitigation behavior directly from environment interaction, without predefined control structures or manual tuning.
- Closed-loop stabilization of an inherently unstable feathered SAFU wing system, with the PPO policy delivering fast, well-damped responses across 5–20 m/s gust conditions and maintaining bounded and smooth actuation.
- Extensive simulation validation, demonstrating that PPO-driven feather actuation inside SAFU enhances gust rejection, improves dynamic robustness, and increases aerodynamic resilience compared with uncontrolled or conventionally controlled setups.

The paper is further prepared as follows: Section 2 describes the design and modeling of the FWFR wing equipped with the SAFU. Section 3 presents the stability analysis, while Section 4 outlines the design of the PPO-based RL controller. Section 5 discusses the simulation results, and Section 6 concludes the paper.

## 2. SAFU Design and Modeling Framework

This study adopts the Festo Smart Bird [31] as the reference flapping-wing platform. The Smart Bird platform has a total wingspan of 2.2 m and an average wing chord of approximately 0.28 m. The proposed SAFU, inspired by avian covert feathers, is implemented as a localized wing-integrated module with approximate spanwise and chordwise dimensions of 12 inches (0.305 m) and 6 inches (0.152 m), respectively. This corresponds to about 14% of the total wingspan and roughly half of the local chord length. This geometric scaling reflects the biological role of covert feathers, which act as localized flow-sensing and flow-modulating elements rather than primary lift-generating structures. Accordingly, the SAFU is designed to influence local aerodynamic behavior in gust-affected wing regions while remaining compatible with the global lift-producing function of the flapping wing.

**Figure 2:**
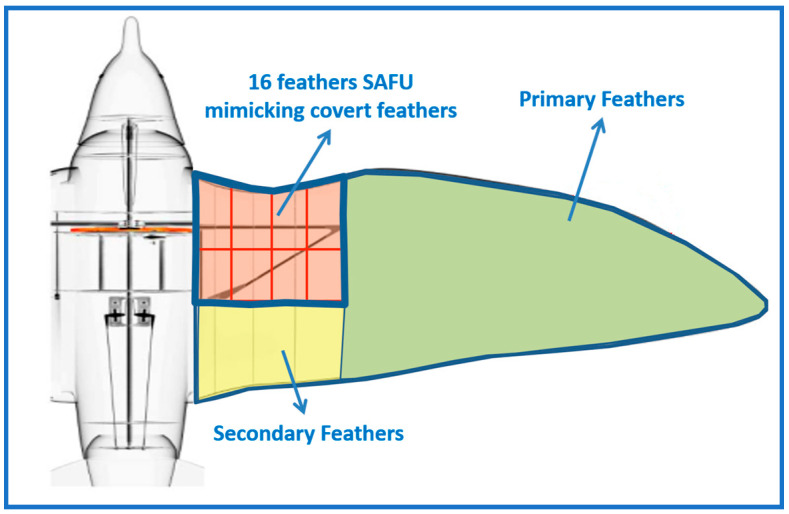
Overall structure of the SAFU-equipped FWFR.

The proposed SAFU architecture incorporates a total of 16 EFs, distributed symmetrically with eight mounted on the upper wing surface and eight on the lower surface. The number of EFs is selected to represent the SAFU region covering approximately 14% of the wingspan in a manner consistent with the typical spatial extent of covert feathers in falcons [28], while maintaining uniform coverage, structural symmetry, and a practical actuator density. Each EF within the bio-inspired SAFU is composed of a flap, mechanical linkage, linear encoder, piezoelectric transducer (PZT) element, voice-coil actuator and a local controller. The voice-coil actuator operates as a linear electromagnetic actuator to provide smooth and fast flap motion suitable for control-oriented gust mitigation. The compact PZTs simultaneously act as high-sensitivity flow sensors and rapid-response actuators. Figure 2 illustrates the overall structure of the SAFU-equipped FWFR, highlighting the placement of the bio-inspired SAFU relative to the primary and secondary wing feathers.

**Figure 3:**
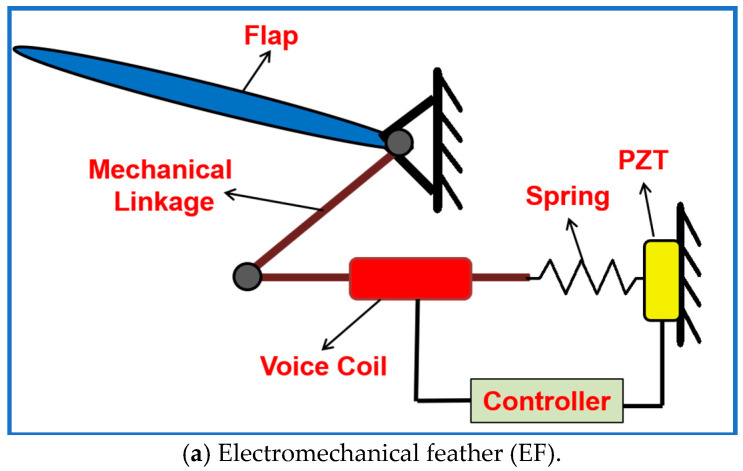

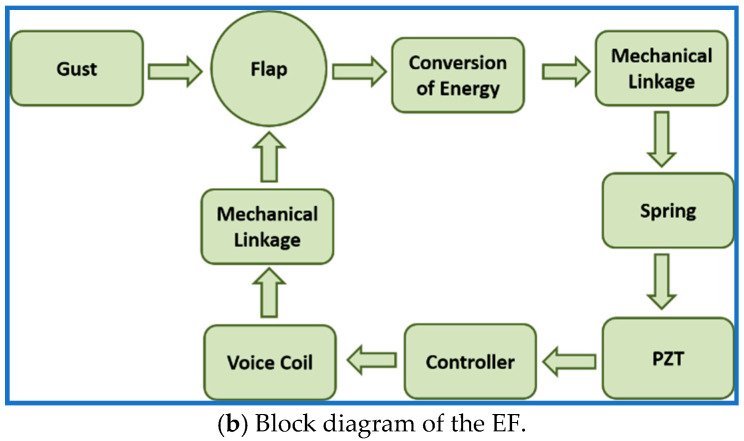
Detailed working diagrams of the EF.

In steady flight, the EFs remain aligned with the wing surface to maintain the airfoil profile. When exposed to strong airflow disturbances, the feathers deflect proportionally to the incident flow, reducing the effective cross-sectional area of the wing and initiating the sensing process. This deflection is transmitted to the PZT, which generates a voltage proportional to the disturbance magnitude. The local controller interprets this sensor signal and commands the voice-coil actuator to further modulate the feather flaps. Through this integrated sensing–actuation pathway, the SAFU provides real-time flow detection and adaptive aerodynamic response, enabling the flapping-wing robot to maintain stability and minimize loading in rapidly varying airflow environments. The structural layout of an individual EF is depicted in Figure 3a, while Figure 3b presents the block-level representation of its operational workflow and energy transfer pathways.

Bond graph is a versatile, energy-based modeling framework capable of describing the dynamic behavior and power interactions of physical systems in a unified manner. Unlike conventional modeling approaches that typically operate within a single domain, bond graphs seamlessly represent mechanical, electrical, hydraulic, thermal, and other energy domains within the same structure. This makes them especially valuable for flapping-wing UAVs, where the coupling between mechanical linkages, actuators, and unsteady aerodynamic loads must be captured with accuracy. Furthermore, bond graphs naturally translate into state-space models, allowing direct integration with modern control design, simulation, and optimization tools. Their modular nature also supports incremental model development, enabling researchers to refine subsystems independently without altering the full system architecture. This capability is particularly useful when introducing bio-inspired components, such as EFs or compliant mechanisms, into an existing FWFR design [32,33].

**Figure 4:**
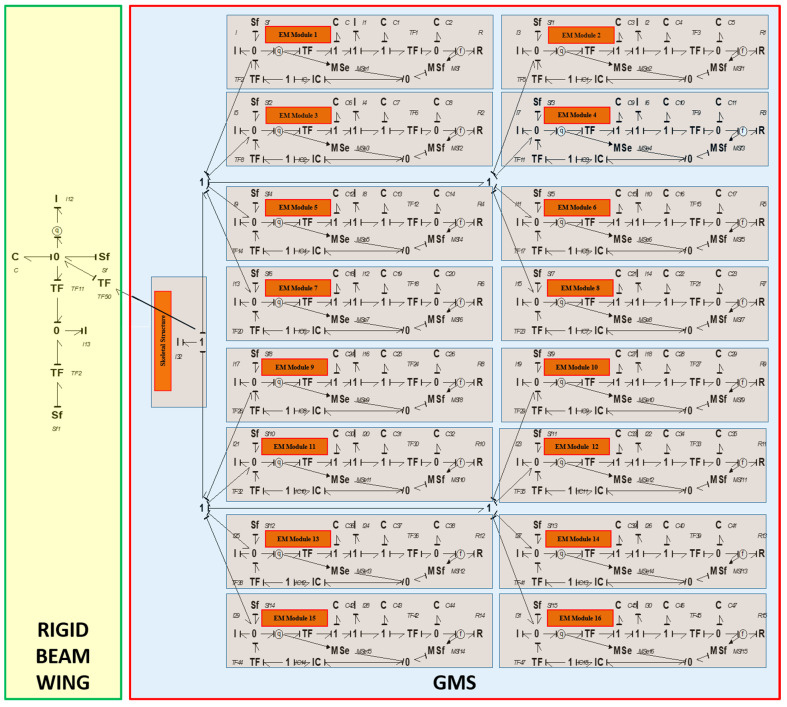
Bond graph model of the proposed SAFU equipped FWFR wing.

Since the proposed SAFU encompasses multiple interacting energy domains—mechanical feather motion, aerodynamic load transfer, and electromechanical actuation: bond-graph modeling becomes an ideal choice, offering an energy-consistent and causally sound way to represent such coupled dynamics. Bond graphs have also been widely applied and experimentally validated in earlier ornithopter and flapping-wing studies [34], reinforcing their credibility as a dependable modeling framework for SAFU-type bioinspired systems. The modeling workflow begins with constructing the bond graph of an individual EF, followed by the rigid-wing model, and ultimately combining these elements into a full bond-graph representation of the wing integrated with all 16 EFs, as depicted in Figure 4. The parameters for all bond graph elements are adopted from the authors’ previous work [29]. For detailed step-by-step model development, readers are referred to the author’s earlier research [29,30].

The mathematical equations for a single EF, derived from the bond graph model depicted in Figure 4, are presented in Equations (1)–(8).

(1) $$\dot{p_{1}}=ic\;\cdot p_{3}+ic\cdot q_{3}$$

(2) $$\dot{q_{1}}=\frac{1}{I_{1}}\;\cdot p_{2}$$

(3) $$\dot{p_{2}}=\frac{ic}{l}p_{3}+\frac{ic}{l}q_{3}-\frac{1}{C}q_{1}-\frac{1}{C_{1}}q_{2}-\frac{m}{C_{2}}q_{4}$$

(4) $$\dot{q_{2}}=\frac{1}{I_{1}}\;\cdot p_{2}$$

(5) $$\dot{p_{3}}=q_{5}$$

(6) $$\dot{q_{3}}=S_{f}-\frac{1}{l\;\cdot \;I_{1}}p_{2}-\frac{1}{I}p_{1}$$

(7) $$\dot{q_{4}}=\frac{m}{I_{1}}p_{2}-\frac{R}{C_{2}}q_{4}$$

(8) $$\dot{q_{5}}=\frac{1}{l\;\cdot \;I_{1}}p_{2}$$

The state variables in above equations consist of *p*_1_, *p*_2_, *p*_3_ which represent the generalized momentum associated with the inertial elements, and *q*_1_, *q*_2_, *q*_3_, *q*_4_ which denote the generalized displacements corresponding to the compliance elements. *I* is mass of feather flap, *S_f_* is gust velocity on flap, *IC* is voice coil actuator compliance and stiffness, *R* is resistance between amplifier and PZT, *I*_1_ is mass of stack, *C*_1_ is PZT spring stiffness, *C*_2_ is PZT equivalent capacitance, *C* is stiffness of spring, *l* is transformer ratio of mechanical linkage. The bond graph model of the full wing equipped with the SAFU, as illustrated in Figure 4, results in a set of 134 differential equations, each representing an energy-storing component, generated using the 20-Sim simulation platform. The state vector $\overset{⃑}{x}\left(t\right)$ includes the generalized momentum of all inertial elements and the generalized displacements of all compliance elements.

To accurately represent the feather-airflow interaction under gust loading, the bond-graph formulation implicitly embeds the fluid–structure interaction (FSI) behavior through aerodynamic effort sources acting on the compliant feather elements. Each EF is modeled as a coupled electromechanical aerodynamic subsystem in which gust-induced aerodynamic forces modify the generalized momenta and displacements of the structural states. This energy-based representation ensures that unsteady aerodynamic effects including load transfer, feather deflection dynamics, and modal coupling between adjacent EFs are captured consistently without requiring explicit CFD coupling. As a result, the full 134-state bond-graph model preserves the dominant FSI pathways responsible for gust attenuation, and these same dynamically influential modes are largely retained in the reduced-order model used for control and learning.

In order to further enhance the aerodynamic representation of the feather-airflow interaction, the aerodynamic effort sources in the bond-graph model are expressed using standard unsteady aerodynamic approximations. For each EF, the quasi-steady lift force generated under gust loading is given by:

(9) $$F_{L}=\frac{1}{2}\rho AC_{L}\left(\alpha \right){\left(V_{g}+\dot{q_{f}}\right)}^{2}$$

where *A* is the feather planform area, ρ is air density, α is the instantaneous feather angle of attack, and *Vg* and $\dot{q_{f}}$ are treated as scalar quantities representing the gust velocity and the effective flap-tip velocity along the local flow direction. For small feather deflections, the lift coefficient is linearized as

(10) $$C_{L}\left(\alpha \right)=C_{L\alpha }\alpha$$

The corresponding aerodynamic moment acting at the feather hinge is

(11) $$M_{a}=F_{L}l_{f}$$

where *l_f_* denotes the moment arm. The gust-induced drag force is similarly modeled as

(12) $$F_{D}=\frac{1}{2}\rho AC_{D}{\left(V_{g}+\dot{q_{f}}\right)}^{2}$$

An equivalent aerodynamic disturbance force is also expressed in terms of gust–structure coupling:

(13) $$F_{g}\left(t\right)=\rho AV_{g}\left(t\right)\dot{q_{f}}$$

In Equations (9)–(13), $q_{f}$ and $\dot{q_{f}}$ denote scalar feather displacement and velocity along the local flow direction. In the reduced-order model, $q_{fi}$ represent the corresponding generalized displacement state variables. In addition, all aerodynamic forces are expressed as scalar quasi-steady equivalents acting along the local feather normal and are incorporated into the bond-graph model as external effort sources, modifying the generalized momenta and displacements of the compliant feather states. This explicit representation provides additional physical insight into the fluid–structure interaction already embedded in the electromechanical subsystem and complements the full 134-state energy-based model.

**Figure 5:**
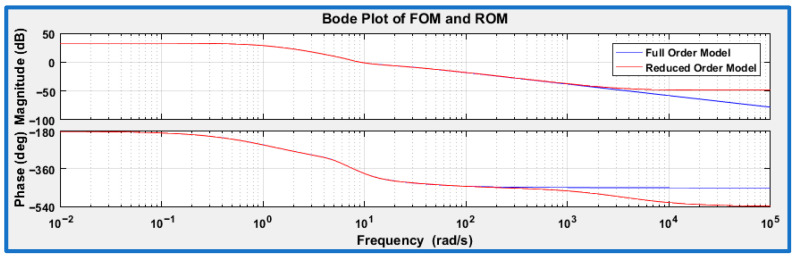
Bode plot comparison of full order model (FOM) vs. reduced order model (ROM).

Balanced truncation is now employed to produce a reduced-order model (ROM) of the SAFU installed FWFR wing system. The method ranks system states using their Hankel Singular Values and removes those with negligible dynamic influence [35]. As shown in Figure 5, the bode plots of the full-order model (FOM) and the ROM match closely across the low and mid frequency ranges, which are most critical for closed-loop operation. Only minor differences appear at high frequencies, where the discarded states contribute very little energy. The maximum deviation in magnitude within the control-relevant range (10^−2^–10^2^ rad/s) is below 1.5 dB, confirming that the approximation error is minimal. Overall, the reduced-order model effectively preserves the key dynamic characteristics of the system and provides adequate fidelity for subsequent simulation and analysis. As a result, a reduced seventh-order model is obtained, comprising seven states corresponding to the generalized displacements of the EF flaps f_1_, f_3_, f_4_, f_6_, f_7_, f_8_, and f_9_ and the state vector is x = [ $q_{f1}\;q_{f3}\;q_{f4}\;q_{f6}\;q_{f7}\;q_{f8}\;q_{f9}]$*^T^*. The resulting state-space representation can be expressed in the general form

(14) $$\begin{array}{c} \dot{\overset{⃑}{x}}\left(t\right)=A\cdot \overset{⃑}{x}\left(t\right)+B\cdot \overset{⃑}{u}\left(t\right)+B_{d}\cdot \overset{⃑}{w}\left(t\right)\;\\ \overset{⃑}{y}\left(t\right)=C\cdot \overset{⃑}{x}\left(t\right)+D\cdot \overset{⃑}{u}\left(t\right) \end{array}$$

where $\overset{⃑}{w}\left(t\right)$ represents wind gust and *B_d_* denotes gust influence. The seventh order state space model of the FWFR wing equipped with SAFU is finally given below:

$$\begin{array}{l} A\;=\;\left[\begin{array}{ccccccc} -8.8 & -2.5 & -3.3 & 1.8 & 5.1 & -3.1 & -6.1\\ -9.9 & -1.2 & -9.1 & 2.05 & 2.6 & -4.2 & -6.7\\ -5.03 & 7.5 & -4.7 & 3.5 & -4.1 & 0 & -7.6\\ -9.3 & 3.3 & -2.9 & -1.5 & -6.5 & 6.1 & 4.9\\ 3.1 & 4.1 & -3 & -1.9 & -8.1 & 3.4 & 6.4\\ 0 & -6.4 & 0 & 7.2 & 3.3 & -0.04 & -2.9\\ -4.4 & 0.4 & -1.4 & 1.2 & -0.7 & -3.5 & -4.03 \end{array}\right]\\ B\;=\;{\left[\begin{array}{ccccccc} 2.9 & 3.2 & 0 & -3.4 & 0.21 & 0 & -0.9 \end{array}\right]}^{T}\\ C\;=\;[\begin{array}{ccccccc} 0.07 & 4.32 & 0 & -0.06 & 0.1 & 0 & 1.9 \end{array}]\;\;D\;=\;[0] \end{array}$$

## 3. Open-Loop Dynamics and Stability Behavior

**Figure 6:**
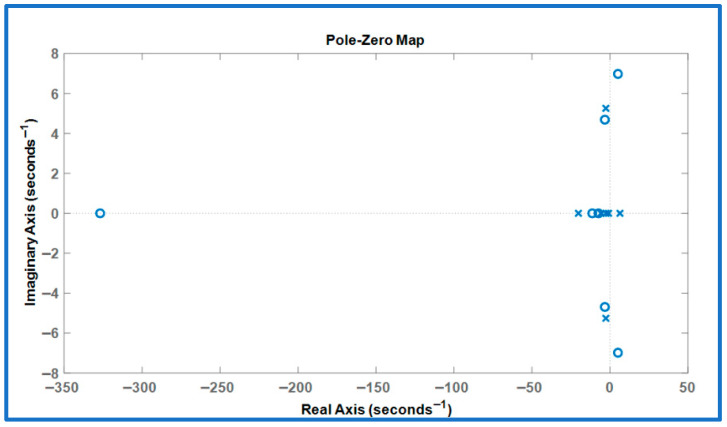
Untrained system’s pole zero plot.

**Figure 7:**
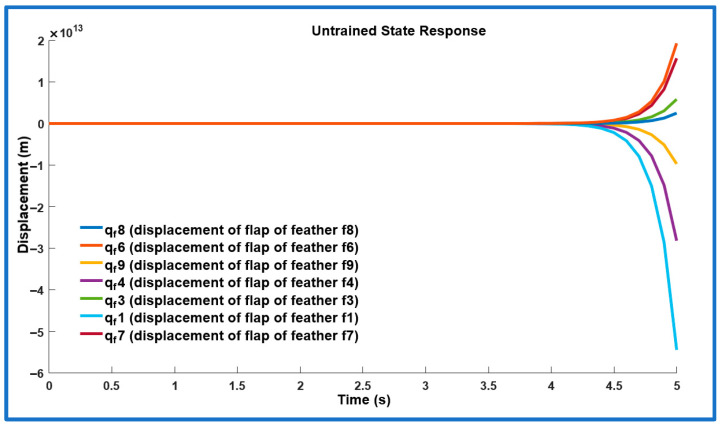
Untrained system’s states response to gust.

Stability analysis of the reduced-order SAFU system is presented in this section. The seven poles of the untrained open loop system are located at: −20, 6.4, −5.8, −0.91, −2.6 ± 5.3j, and −2.6. The presence of a right-half-plane pole at 6.4 indicates that the system is inherently unstable. This is further confirmed by the untrained system’s pole zero plot shown in Figure 6. Additionally, Figure 7 illustrates the unstable state response of the open-loop system when subjected to gust disturbances. This instability underscores the need for design of a PPO-based RL controller for SAFU equipped FWFR wing to ensure attitude stability under both nominal weather conditions and gust disturbances.

## 4. PPO-Based RL Controller Design

This section presents the PPO-based RL controller developed for the SAFU-equipped FWFR wing model. The control architecture is entirely model-free and implemented within a custom OpenAI Gym environment that interfaces directly with the seven-state reduced-order dynamics of SAFU described earlier. The PPO agent is trained to learn an optimal continuous control policy capable of attenuating gust-induced disturbances by regulating the actuator force that drives the bioinspired feather mechanism. Through iterative interaction with the simulated environment, the agent autonomously shapes its policy without relying on gain scheduling, online parameter estimation, or explicit disturbance modeling. Consequently, the learned PPO controller adapts naturally to variations in aerodynamic loading and offers a robust, data-driven alternative to conventional model-based control strategies.

### 4.1. Proximal Policy Optimization (PPO)

**Figure 8:**
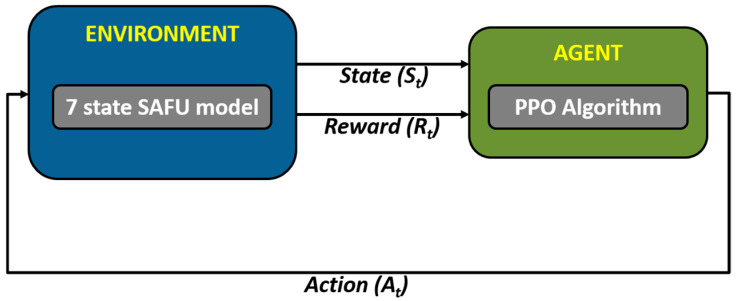
Minimal PPO RL architecture used for SAFU control.

In this study, the RL framework is based on the PPO algorithm, which is a stochastic actor-critic method well suited for continuous control problems involving nonlinear and disturbance-driven systems such as the SAFU-equipped FWFR. PPO is selected in this study due to its stable learning behavior, bounded policy updates, and suitability for continuous control of nonlinear and disturbance-driven systems, enabling smooth and reliable actuation of the SAFU mechanism without extensive hyperparameter tuning. The PPO control architecture used in this research is illustrated in block diagram given as Figure 8. As with most policy-gradient approaches, the learning problem is formulated as a Markov Decision Process (MDP) defined by the tuple [36,37]:

(15) M = (S, A, T, r, γ)

where *S* and *A* denote the state and action spaces, *T*(*s_t_*_+1_ | *s_t_*, *a_t_*) denote the state-transition dynamics, *r*(*s_t_*, *a_t_*) is the reward, and *γ* ∈ (0, 1) is the discount factor that determines the contribution of future rewards. The stochastic policy *π_θ_*(*a_t_*/*s_t_*), parameterized by the actor network with weights *θ*, is optimized to maximize the expected discounted return:

(16) $$J\left(\theta \right)=E_{\pi_{\theta }}\left[\sum_{k=0}^{\infty }\gamma^{k}\;r\left(s_{t+k},\;a_{t+k}\right)\right]$$

PPO improves policy optimization by restricting excessively large policy updates through a clipped surrogate objective. The objective used during training is given by [36,37]:

(17) $$L^{\mathrm{CLIP}}\left(\theta \right)=E\left[\min\left(r_{t}\left(\theta \right){\hat{A}\;}_{t}\;,|clip\left(r_{t}\left(\theta \right),\;1-\epsilon ,\;1+\epsilon \right)\;{\hat{A}\;}_{t}\;\right)\right]$$

where

(18) $$r_{t}\left(\theta \right)=\frac{\pi_{\theta }\left(a_{t}|s_{t}\right)}{\pi_{\theta_{\mathrm{old}}}\left(a_{t}|s_{t}\right)}$$

is the likelihood ratio, *ϵ* is the clipping parameter, and ${\hat{A}\;}_{t}$ is the generalized advantage estimator (GAE) defined as [38]:

(19) $${\hat{A}\;}_{t}=\sum_{l=0}^{\infty }{\left(\gamma \lambda \right)}^{l}\left(r_{t+l}+\gamma V\left(s_{t+l+1}\right)-V\left(s_{t+l}\right)\right)$$

The critic network, parameterized by *ϕ*, approximates the state-value function *V_ϕ_* (*s_t_*) and is trained by minimizing the mean-squared error: [36,37]:

(20) $$L^{\mathrm{VF}}\left(\phi \right)=E\left[{\left(V_{\phi }\left(s_{t}\right)-R_{t}\right)}^{2}\right]$$

where,

(21) $$R_{t}={\hat{A}\;}_{t}+V_{\phi }\left(s_{t}\right)$$

is the estimated return. To prevent destructive parameter updates, PPO alternates between data collection and multiple epochs of minibatch optimization using the clipped objective. This ensures stable and conservative policy improvement while maintaining sample efficiency.

The actor–critic architecture used in this work consists of two neural networks: (1) a stochastic actor that outputs the mean and variance of a Gaussian control distribution, and (2) a critic that approximates the state-value function. Both networks receive the seven-dimensional state vector as input and employ two fully connected hidden layers (64 × 64 neurons) with ReLU activation. The stochastic policy enables smooth and continuous actuation of the SAFU mechanism, while the critic provides stable value estimation required for GAE computation. Through repeated interaction with the FWFR environment, the PPO algorithm converges toward an optimal control policy that minimizes gust-induced disturbances and enforces smooth force modulation across the wing-feather linkage.

### 4.2. Control Task

The control task in this study is to enable the SAFU-equipped FWFR wing to maintain stable aerodynamic behavior in the presence of externally applied gust disturbances, while ensuring that the applied actuator forces remain within safe and efficient operating limits. The PPO agent is trained to minimize deviation across all system states, suppress overshoot, and guide the seven-state dynamics toward their equilibrium configuration under gust levels representative of magnitudes up to 20 m/s. Each training episode begins from a slightly perturbed initial condition and subjects the system to a step-type gust input that excites all dynamic modes of the reduced-order model. The controller is required to counter these disturbances promptly and restore the wing-feather mechanism to steady behavior without violating actuator constraints. Successful learning is observed through smooth convergence of all states to their nominal values within roughly 1.5 s, with control forces consistently remaining below approximately 0.8 N, indicating effective disturbance rejection and energy-efficient adaptation.

### 4.3. PPO Learning Environment

We develop a dedicated training environment to pair the PPO agent with the reduced-order dynamics of the SAFU-integrated FWFR wing. Rather than relying on preset control laws or analytical tuning, the environment serves as an interactive simulation layer through which the agent continuously probes the system, applies actions, and refines its policy. Built in Python and structured according to the OpenAI Gym interface, it embeds the full state-space model of the SAFU-integrated FWFR wing, where gust disturbances enter as external forcings and the control action represents the actuator effort delivered to the flow-control unit. At every simulation step, the agent receives the normalized seven-state observation, issues a continuous control command, and is evaluated through a reward that balances convergence quality, overshoot suppression, control smoothness, and physical constraint satisfaction. This gym-compatible design integrates seamlessly with a PyTorch 1.10 PPO implementation, enabling consistent training, benchmarking, and policy assessment across a range of gust scenarios.

The reduced-order model used inside the environment provides a fast yet dynamically representative approximation of the full SAFU equipped FWFR wing behavior. Although PPO does not directly utilize this model for analytical control derivation, it acquires its policy entirely through repeated interaction with these simulated dynamics. By condensing the original high-dimensional formulation into seven dominant states, the environment preserves the essential mechanical and aerodynamic coupling while remaining computationally efficient for long-horizon learning episodes.

The observation space consists of a 7-dimensional continuous vector representing the normalized generalized displacements of the seven dominant SAFU feather states. These states encode the reduced-order aero-electromechanical dynamics that the PPO agent uses to infer the current aerodynamic condition. The action space is one-dimensional and continuous, corresponding to the actuator force applied to the EF mechanism. This control signal is bounded within ±0.8 N to ensure physically realistic and safe feather actuation.

### 4.4. Reward Design

The reward structure we use for PPO training is composed of four smoothly varying penalty terms that guide the controller toward stable and efficient regulation of the SAFU system. At each simulation step, the agent receives a scalar reward formed as the negative weighted sum of key performance measures. The first component penalizes the overall state magnitude through a quadratic term, promoting rapid convergence of all seven states toward their equilibrium values. A second quadratic term penalizes the instantaneous control input, encouraging energy-efficient actuation and preventing unnecessarily large forces. The aerodynamic response of the feather mechanism is regulated through a proportional penalty on the absolute value of the output *y*, discouraging excessive feather-force deviations. To avoid abrupt or chattering actuation, a smoothness term based on the squared difference between the current and previous inputs, (*u* − *u_prev_*)^2^, is included. Here, *u_prev_* denotes the control command applied at the preceding time step and enables the reward to penalize rapid variations in the actuator signal, thereby inducing physically consistent and mechanically safe force modulation. The resulting reward employed during training is expressed as

(22) $$r=-\left(0.5\sum_{i=1}^{7}|x_{i}{|}^{2}+0.05\;u^{2}+0.5\left|y\right|+0.1{\left(u-u_{\mathrm{prev}}\right)}^{2}\right)$$

The relative weights of the reward terms are determined through an iterative reward-shaping process during controller development, with the primary objective of ensuring stable convergence of all seven states under gust excitation while maintaining bounded and smooth actuation. The selected weighting represents a balanced configuration that consistently yielded stable learning behavior and robust closed-loop performance across multiple training runs and gust conditions. This formulation provides clear optimization guidance, enabling the PPO agent to achieve smooth state convergence, restrained actuator effort, controlled feather-force behavior, and stable, low-oscillation actuation throughout the learning process. Moreover, the detailed reward-weight sensitivity analysis forms part of future work.

### 4.5. Training Hyperparameters

We implement the PPO controller using Adam optimizer with separate learning rates for the policy and value networks to ensure balanced convergence between policy updates and value estimation. Key training parameters including the discount factor γ, GAE parameter λ, policy-clipping threshold ϵ, number of update epochs, and rollout horizon—are tuned to maintain stable learning without premature policy collapse. These hyperparameters govern the rate of policy improvement and the smoothness of advantage estimation during training. The complete list of PPO controller hyperparameters used in this study is summarized in Table 1.

### 4.6. Computational Framework for PPO Learning

We carry out the PPO training process for over 500 episodes, with each episode running up to 500 simulation steps under a fixed integration time step of 0.01 s. Rather than relying on stored experience, we generate each rollout on-policy and use immediately for policy and value-function updates. We implement training in Python 3.10 using the PyTorch framework and execute on a Windows 10 workstation equipped with an Intel^®^ Core™ i7 processor (2.9 GHz) and 16 GB of RAM. The average computation time per episode is approximately 3–4 s, and the reward signal typically stabilizes after 270 episodes. Following training, we employ the learned actor network for closed-loop simulations to assess state convergence and control behavior under different gust magnitudes.

To clarify the scope of the PPO-based controller, several practical considerations are noted that guide future refinement of the proposed framework. The training environment employs a reduced seventh-order linear model, which captures the dominant dynamics of the SAFU-equipped FWFR wing but does not explicitly represent all nonlinear aerodynamic effects. Gust disturbances are modeled using step-type inputs, which serve as conservative and repeatable benchmarks for evaluating disturbance rejection and transient stability, although real atmospheric conditions may exhibit more complex and stochastic variability. In addition, actuator saturation effects and sensor noise are not explicitly included in the present model. Incorporating higher-fidelity aerodynamic representations, more realistic gust profiles, and extended simulation conditions is an important planned future work to further enhance the robustness and practical applicability of the learned control policy.

## 5. Simulation Results and Discussions

**Figure 9:**
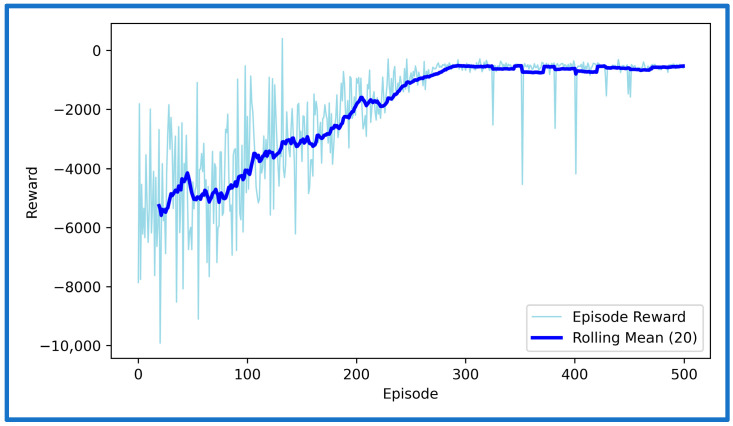
Training reward vs. episode of the PPO agent.

**Figure 10:**
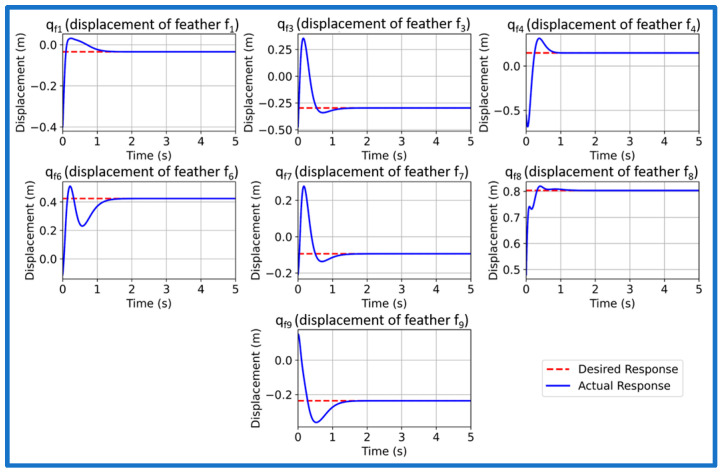
PPO controlled state response at 5 m/s gust.

**Figure 11:**
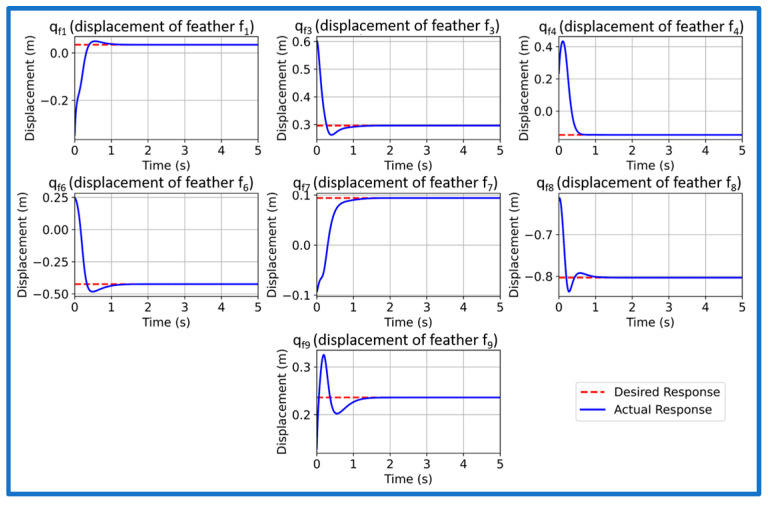
PPO controlled state response at 10 m/s gust.

**Figure 12:**
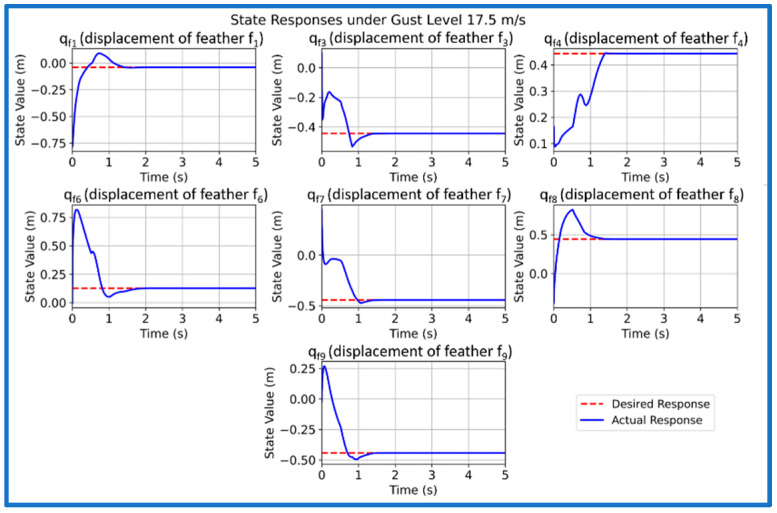
PPO controlled state response at 15 m/s gust.

**Figure 13:**
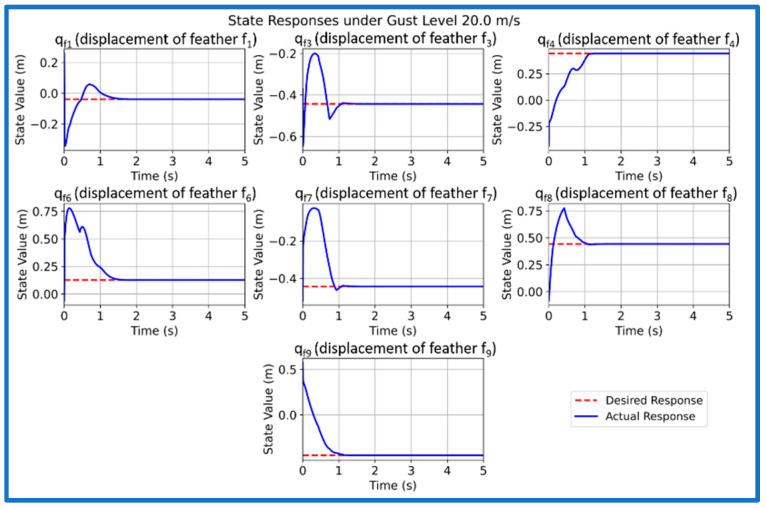
PPO controlled state response at 20 m/s gust.

We now simulate and analyze the developed SAFU equipped FWFR wing model and its PPO-based RL controller in this section. Figure 9 illustrates the training reward evolution of the PPO-based controller with episodes. We see a high variability during early episodes that reflects exploration, while the rolling mean reward converges to a stable level, indicating consistent and successful policy learning. After successfully concluding PPO training in the custom OpenAI-Gym environment, we now extract and deploy the learned actor policy for performance validation. The evaluation applies the optimized policy trained at 15 m/s gust to unseen gust magnitudes of 5, 10, 15, and 20 m/s to examine generalization and robustness. Each simulation introduces a step-gust disturbance and records the time-domain evolution of all seven states, with a target settling time below approximately 1.5 s. Figure 10, Figure 11, Figure 12 and Figure 13 present the resulting state trajectories for all gust levels. The overall behavior of the SAFU-equipped FWFR wing under these aerodynamic conditions is summarized below.

Under the lowest gust magnitude of 5 m/s, the PPO controller produces a highly damped response with near-instant rise times under 0.49 s and settling of all states within 1.35 s. The mild aerodynamic loading results in smooth, clean trajectories with virtually no oscillatory activity. All seven states converge rapidly, confirming that the PPO policy preserves stability and accurate state regulation even under weak disturbances. For the moderate 10 m/s gust, the controller maintains fast transient behavior with rise times below 0.45 s and settling times between 0.36 and 1.22 s. The PPO agent suppresses small initial deviations without allowing secondary oscillations to develop. The overall damping remains consistent across the system, demonstrating that the learned control law adapts well to increased aerodynamic coupling among the feathers. At the nominal 15 m/s gust used during training, the PPO policy yields the most balanced and uniform response. Rise times remain below 0.43 s and settling occurs within 0.72–1.10 s, matching the intended design performance. The transient peaks of all states appear well-aligned, reflecting that the controller has internalized the optimal compromise between fast convergence and moderated control effort at its trained operating point. When subjected to the strongest gust of 20 m/s, the controller still delivers bounded and stable trajectories. Rise times remain low, below 0.48 s, while settling times extend mildly to 1.18 s due to higher aerodynamic loading. Although transient excursions are slightly larger, the PPO agent consistently drives all states back to equilibrium without divergence, overshoot growth, or sustained oscillations. This indicates robustness at the upper edge of the disturbance envelope.

Across all four gust levels, the PPO-based controller exhibits a uniform stabilization pattern. While settling times vary modestly with gust intensity, the qualitative structure of each trajectory remains unchanged: smooth rise, rapid decay of transients, and clean convergence. This consistency suggests that the policy learns a generalizable mapping between gust-induced aerodynamic perturbations and optimal corrective actions, rather than memorizing dynamics specific to the training case. The absence of divergence, oscillatory drift, or steady-state deviation across the full gust range confirms that the PPO-trained SAFU controller achieves the key characteristics required for real-world gust-mitigation: robustness, adaptability, and reliable generalization.

**Figure 14:**
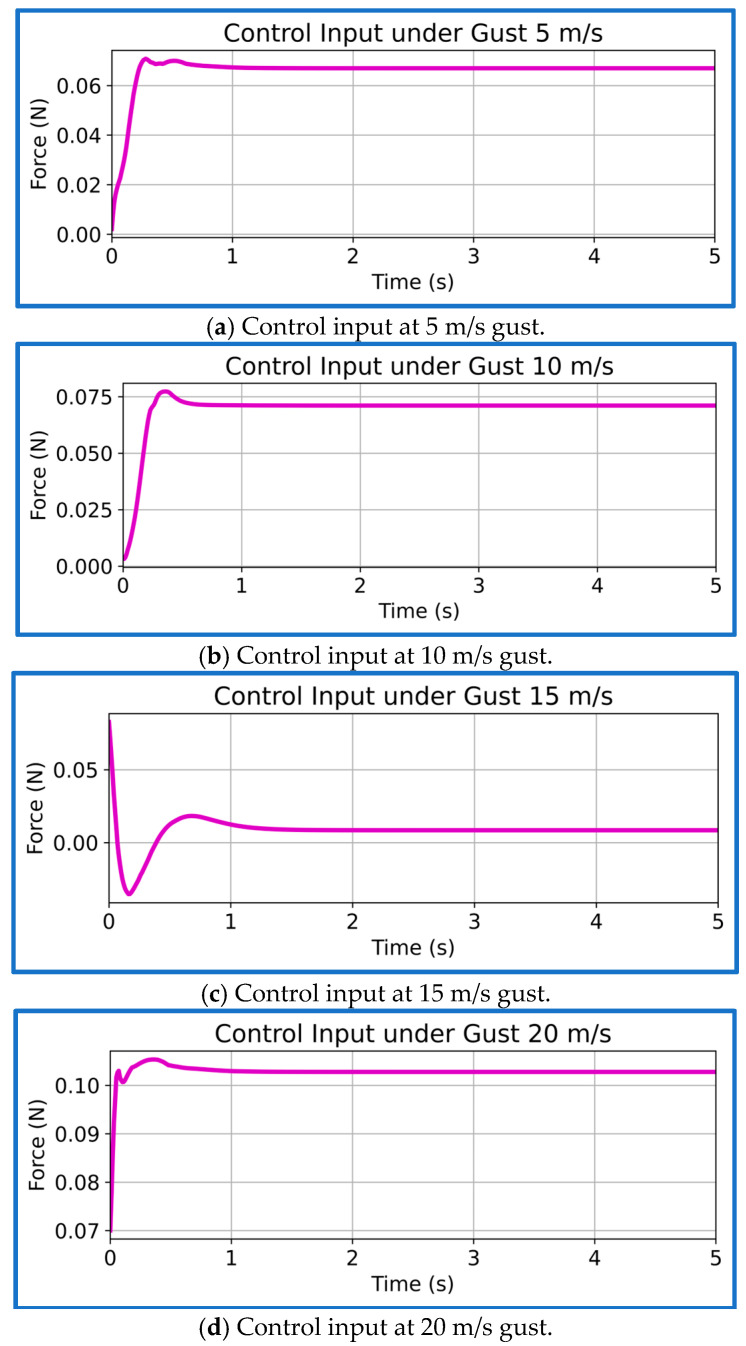
Control input plots of PPO controller at different gust speeds.

We present the corresponding PPO control-input profiles for all four gust scenarios in Figure 14a–d. Across the entire range, the control effort remains smooth, stable, and well within the actuator limits. For the mild 5 m/s gust, the actuator produces only gentle, low-amplitude corrections, reflecting the small disturbance energy. At the 10 m/s gust, the modulation increases slightly but stays clean and well-damped, without any aggressive switching. For the 15 m/s training gust, the control signal becomes the most consistent and near-periodic, showing that the learned policy has internalized an efficient balance between disturbance rejection and energy usage. Under the highest 20 m/s gust, the PPO controller increases force just enough to counter the stronger loading, yet the input still converges quickly after the initial transient and remains strictly inside the ±0.8 N bound. Overall, the control-input trends confirm that the reward structure, particularly the quadratic effort penalty and action-limit constraint, successfully enforces smooth, bounded, and energy-aware actuation across all gust intensities.

The rise times and settling times of all seven SAFU system states under the four gust levels (5, 10, 15, and 20 m/s) are reported in Table 2. The results show that the PPO controller maintains consistent and well-damped transient behavior across all gust intensities, with every state settling within approximately 0.36–1.35 s and no steady-state deviation. Small differences in rise time reflect the aerodynamic coupling between neighboring feathers, whereas the uniformly fast settling across all conditions confirms the robustness and adaptability of the learned PPO policy.

**Figure 15:**
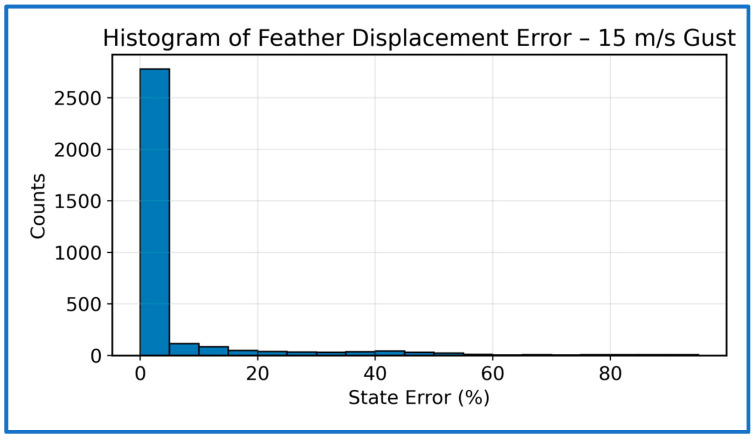
Histogram of normalized state errors at 15 m/s gust.

The following subsection examines the error characteristics of the PPO-trained SAFU controller under the 15 m/s gust condition. Figure 15 shows the distribution of feather-displacement errors. Most samples fall within a small error band, with a dominant cluster near 0–10%, reflecting strong steady-state regulation. A gradually decaying right-tail corresponds to the brief transient deviations immediately after gust onset. These excursions fade quickly and do not persist, indicating effective suppression of high-frequency disturbances. The absence of wide tails or secondary peaks further confirms stable and consistent PPO control throughout the simulation.

**Figure 16:**
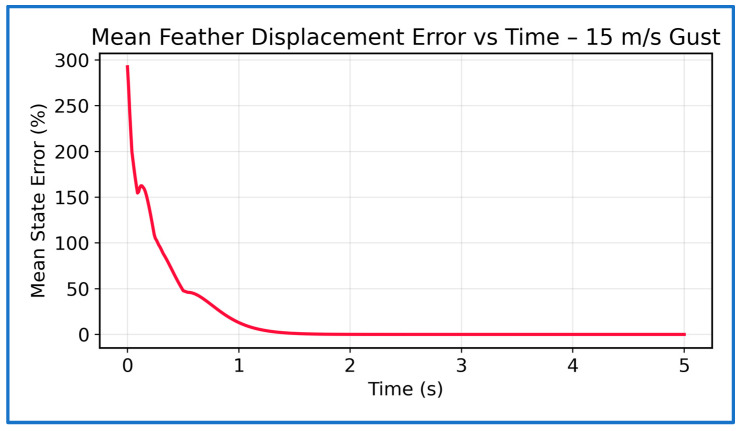
Mean state error vs. time at 15 m/s gust.

Figure 16 shows the temporal evolution of the mean displacement error across all seven states. Following gust injection, the error exhibits a brief transient peak before decaying rapidly in an exponential-like manner, falling below 5% within about 1 s. After this point, only small, bounded fluctuations remain, indicating effective gust rejection and fast convergence. The lack of overshoot or sustained oscillations further confirms the stability and smoothness of the PPO control policy. Together, these figures show that the PPO-based SAFU controller delivers fast transient suppression, high steady-state accuracy, and robust convergence under strong gusts. The histogram confirms that errors remain tightly clustered, while the time-series plot shows rapid decay from the initial transient to near-zero within about one second. Overall, the results demonstrate stable and reliable PPO control, keeping displacement errors well within acceptable aerodynamic limits even in aggressive gust conditions.

**Figure 17:**
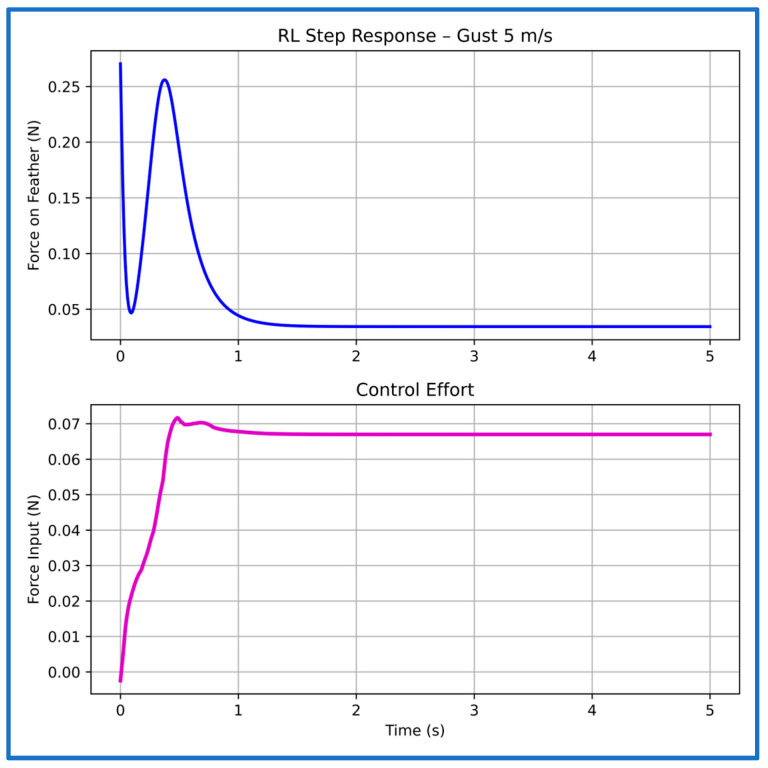
Step response and control effort of the PPO controller at 5 m/s gust speed.

**Figure 18:**
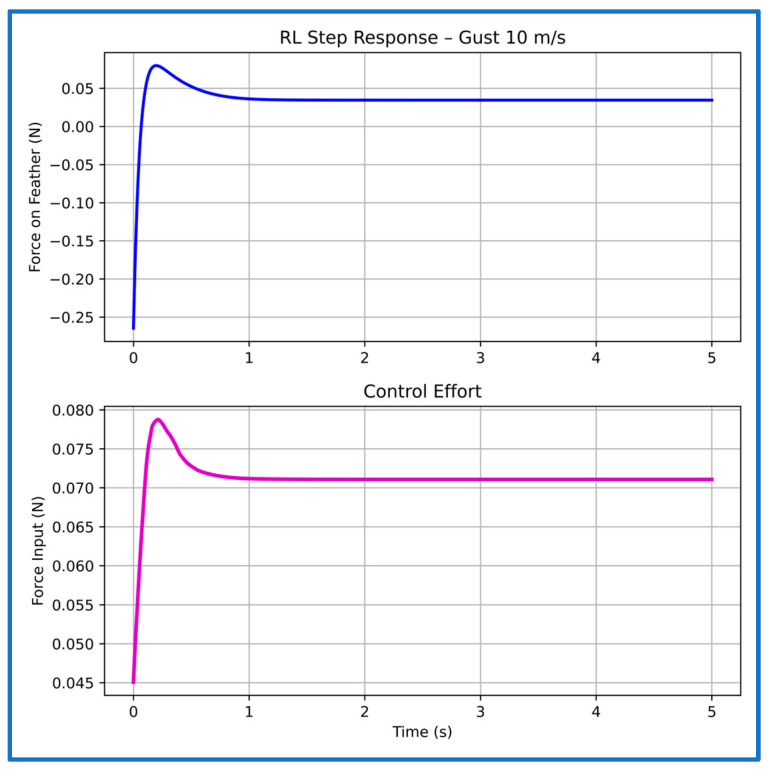
Step response and control effort of the PPO controller at 10 m/s gust speed.

**Figure 19:**
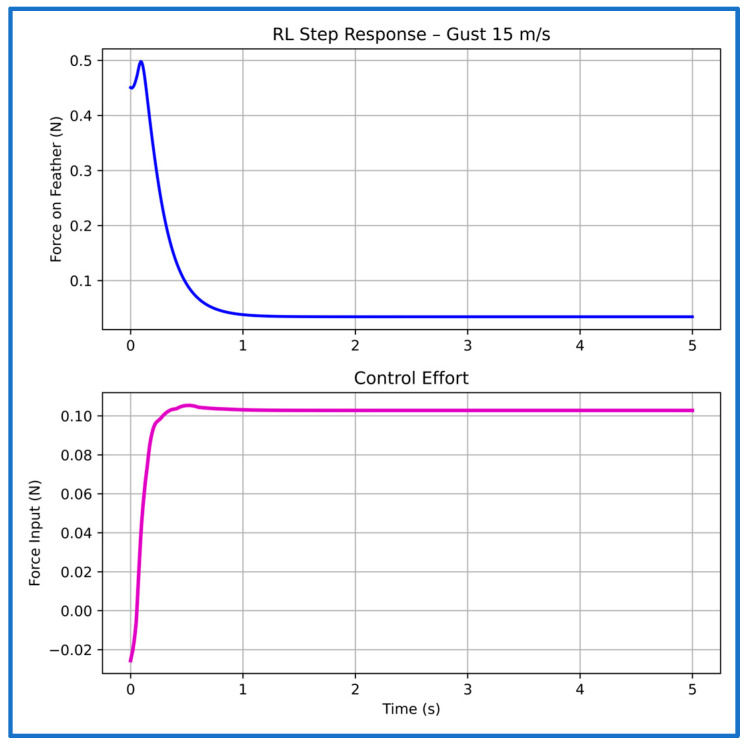
Step response and control effort of the PPO controller at 15 m/s gust speed.

**Figure 20:**
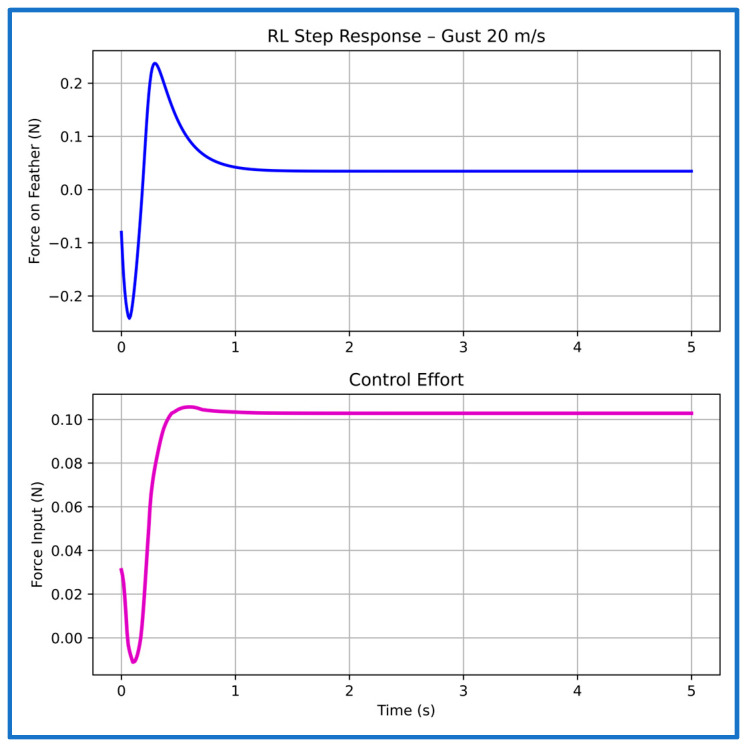
Step response and control effort of the PPO controller at 20 m/s gust speed.

We now present the representative step responses and corresponding control efforts of the PPO-based controller under four gust conditions in Figure 17, Figure 18, Figure 19 and Figure 20. Under the 5 m/s gust (Figure 17), the controller rapidly attenuates the transient force, producing a smooth, well-damped response with no overshoot and settling in approximately 1.16 s. The control effort remains comfortably within the ±0.8 N actuator limit, indicating efficient regulation under mild aerodynamic loading.

For the 10 m/s gust (Figure 18), the system exhibits a very fast rise of 0.02 s and settles in about 1.02 s, with the actuator force staying bounded and cleanly modulated. This shows that PPO adapts effectively to the increased disturbance without introducing oscillations or excessive energy use.

At the 15 m/s training gust (Figure 19), the controller maintains similarly fast dynamics, with a rise time of 0.06 s and settling around 1.01 s. The trajectory converges smoothly with negligible overshoot, reflecting that the learned policy has internalized an efficient mapping between aerodynamic feedback and actuator response.

Even under the strongest 20 m/s gust (Figure 20), the response remains stable and well-controlled, with a rise time of 0.05 s and settling within 1.20 s. No divergence or high-frequency oscillations appear, and the actuator output adjusts smoothly and stays well inside the force limits, confirming effective generalization to severe gust conditions.

Across all gust levels, the PPO controller consistently delivers rapid rise, tightly clustered settling times to less than 1.2 s, and full suppression of oscillations. These results contrast sharply with the unstable untrained SAFU response (Figure 6 and Figure 7) and demonstrate that the learned PPO policy stabilizes the previously divergent system and provides robust gust-rejection capability across the entire tested range. Table 3 summarizes the step-response characteristics.

**Figure 21:**
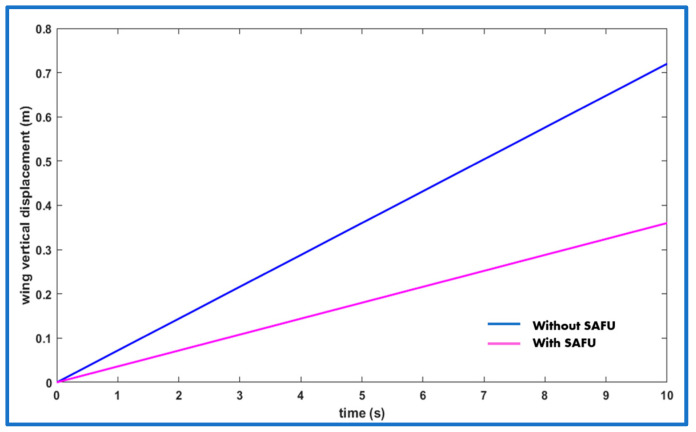
Vertical displacement of the FWFR wing subjected to a gust of 15 m/s.

Figure 21 presents the vertical displacement response of the FWFR wing under a 15 m/s gust for two cases: without a SAFU and with a SAFU governed by the PPO-based RL controller. The results indicate that the actuation of the SAFU’s EFs mitigates nearly half of the gust-induced disturbance, while the PPO-based controller further improves the response by delivering faster stabilization, greater robustness, and more efficient control action. This successfully certifies the efficiency of the proposed SAFU-augmented FWFR wing controlled with proposed PPO-based RL framework in the tackling of gusts.

The comparison of the proposed PPO-based RL control approach with state of the art is presented in Table 4. The comparative analysis in Table 4 further validates the effectiveness of the proposed PPO-based controller. Unlike prior studies that demonstrated gust tolerance limited up to 7 m/s, the proposed method maintains stable performance under gusts up to 20 m/s, representing the highest disturbance level reported among the compared works. While existing approaches such as strain-based sensing with RL [13] and hybrid RL–PD [21] achieve reliable recovery within moderate wind ranges of up to 7 m/s, and classical LQR [24] stabilizes only up to 3 m/s, the proposed method delivers up to 50% gust rejection at gusts up to 20 m/s with a settling time comparable to or better than earlier controllers. This indicates that the learned policy not only extends the disturbance-handling envelope significantly but also preserves fast convergence, thereby demonstrating a substantial advancement over state-of-the-art FWFRs’ gust-mitigation strategies.

The results shown in Table 4 are based on reported results from the literature and reflect the general gust-response behavior of ornithopter and flapping-wing platforms. The proposed SAFU-equipped FWFR uses a new bio-inspired sensing and actuation mechanism that does not exist in earlier designs; therefore, a direct real-time comparison under the same environment and initial conditions is not possible without changing the structure of the other systems. The purpose of this comparison is to place the achieved gust-rejection performance within the overall range of results reported for ornithopter-based platforms.

## 6. Conclusions

This work presented a falcon-inspired sensing and actuation feather unit (SAFU) with electromechanical feathers (EFs) governed by a Proximal Policy Optimization (PPO) reinforcement learning (RL) controller for gust mitigation in flapping-wing flying robots (FWFRs). Using a reduced-order bond-graph model to represent the coupled wing–feather dynamics, the PPO agent learned effective gust-rejection behavior directly from interaction with the environment, without predefined control laws. The trained controller stabilized the inherently unstable open-loop SAFU and achieved fast, well-damped responses across gusts of 5–20 m/s, with rise times below 0.5 s and settling times below 1.4 s. Control inputs remained smooth and bounded within actuator limits, and high-frequency disturbances were effectively suppressed. These results demonstrate that PPO-driven feather actuation in SAFU equipped FWFR offers a robust, adaptive, and energy-aware gust-mitigation strategy for next-generation bio-inspired ornithopters.

In the future, we aim to progressively improve model realism by incorporating CFD-based aerodynamic analyses and evaluating controller performance under more diverse and stochastic gust environments. Higher-fidelity simulations will be used to examine the interaction between unsteady airflow and the electromechanical feathers. The influence of feather number and spatial placement on gust alleviation will also be assessed. In addition, hardware-in-the-loop experiments and small-scale prototype testing are planned to study actuator dynamics, sensing noise, and communication delays. Finally, the adaptability of the trained PPO controller to variations in wing and feather configurations, as well as its robustness to model uncertainties and stochastic and Dryden turbulence, will be examined to support practical deployment.

## Figures and Tables

**Table 1:** Hyperparameters for the PPO controller.

Description	Symbol	Value
Actor learning rate	α_a_	3 × 10^−4^
Critic learning rate	α_c_	10^−3^
Discount factor	γ	0.99
GAE parameter	λ	0.95
PPO clipping threshold	ϵ	0.2
Number of PPO update epochs	-	2
Hidden layer dimension	l	64 × 64
Number of state variables	n_x_	7
Number of control outputs	n_u_	1
Maximum steps per episode	t	500
Total training episodes	K	500

**Table 2:** Rise time (RT) and settling time (ST) of individual feather state responses under different gust magnitudes for the PPO-based RL controller.

States {Displacement (m) of Feathers}	5 m/s Gust		10 m/s Gust		15 m/s Gust		20 m/s Gust	
	RT (s)	ST (s)	RT (s)	ST (s)	RT (s)	ST (s)	RT (s)	ST (s)
q_f1_	0.01	1.07	0.01	0.83	0.01	0.78	0.01	0.73
q_f3_	0.01	1.20	0.01	0.69	0.01	1.08	0.06	0.98
q_f4_	0.02	0.78	0.06	0.57	0.07	0.73	0.01	0.51
q_f6_	0.16	1.34	0.45	1.07	0.15	1.03	0.07	0.96
q_f7_	0.14	1.30	0.01	1.22	0.01	1.05	0.01	1.02
q_f8_	0.28	0.42	0.22	0.36	0.43	0.72	0.48	0.61
q_f9_	0.49	1.35	0.14	1.09	0.05	1.10	0.01	1.18

**Table 3:** Characteristics of the PPO controller’s step plots at multiple gust speeds.

Gust Speed (m/s)	Rise Time (s)	Settling Time (s)
5	0.01	1.16
10	0.02	1.02
15	0.06	1.01
20	0.05	1.20

**Table 4:** Comparison of the proposed PPO-based RL SAFU controller with the literature.

Study	Technique	Type of Disturbance	Disturbance Rejection Results	Settling/ Convergence Time
Kim et al. [13]	Strain-based sensing + RL	3–7 m/s	Adaptive and stable flight	Converges to the target within approximately 5 s
Yu et al. [21]	Reinforcement Learning and Hybrid RL–PD	Extreme attitudes (flips, aggressive maneuvers)	Decreased angular accelerations and stable recovery	≈1.5 s
Bhatia et al. [24]	LQR	Up to 3 m/s	Less oscillations and stabilized attitude	Converges within 1.8 s
Zheng et al. [22]	Model Predictive Control	Up to 0.25 m/s	Rapid convergence to reference	Less than 3 s
Proposed	Bio-inspired SAFU with PPO-based RL control	Up to 20 m/s	Up to 50% gust rejection	All states settle within 1.4 s

## Data Availability

The data that support the findings of this study are available from the corresponding author, upon reasonable request.
